# Evaluating the Need for Routine COVID-19 Testing of Emergency Department Staff: Quantitative Analysis

**DOI:** 10.2196/20260

**Published:** 2020-12-03

**Authors:** Yuemei Zhang, Sheng-Ru Cheng

**Affiliations:** 1 Department of Anesthesiology and Pain Medicine University of Washington Seattle, WA United States

**Keywords:** infectious, diseases, COVID-19, modeling, policy, emergency medicine, healthcare, health policy, screening tests, surveillance screening

## Abstract

**Background:**

As the number of COVID-19 cases in the US continues to increase and hospitals experience shortage of personal protective equipment (PPE), health care workers have been disproportionately affected. However, since COVID-19 testing is now easily available, there is a need to evaluate whether routine testing should be performed for asymptomatic health care workers.

**Objective:**

This study aimed to provide a quantitative analysis of the predicted impact that regular testing of health care workers for COVID-19 may have on the prevention of the disease among emergency department patients and staff.

**Methods:**

Using publicly available data on COVID-19 cases and emergency department visits, as well as internal hospital staffing information, we developed a mathematical model to predict the impact of periodic COVID-19 testing of asymptomatic staff members of the emergency department in COVID-19–affected regions. We calculated various transmission constants based on the Diamond Princess cruise ship data, used a logistic model to calculate new infections, and developed a Markov model based on the average incubation period for COVID-19.

**Results:**

Our model predicts that after 180 days, with a transmission constant of 1.219e-4 new infections/person^2^, weekly COVID-19 testing of health care workers would reduce new health care worker and patient infections by approximately 3%-5.9%, and biweekly testing would reduce infections in both by 1%-2.1%. At a transmission constant of 3.660e-4 new infections/person^2^, weekly testing would reduce infections by 11%-23% and biweekly testing would reduce infections by 5.5%-13%. At a lower transmission constant of 4.067e-5 new infections/person^2^, weekly and biweekly COVID-19 testing for health care workers would result in an approximately 1% and 0.5%-0.8% reduction in infections, respectively.

**Conclusions:**

Periodic COVID-19 testing for emergency department staff in regions that are heavily affected by COVID-19 or are facing resource constraints may significantly reduce COVID-19 transmission among health care workers and previously uninfected patients.

## Introduction

Although COVID-19 originated as a small cluster of cases restricted to Wuhan, China, in November and December 2019, SARS-CoV-2, the causative virus, has rapidly spread across the globe since then. On March 11, 2020, the World Health Organization officially declared COVID-19 as a pandemic [1]. In the United States, the number of confirmed COVID-19 cases spiked from only 1 case on January 20, 2020, to 6,244,970 confirmed cases and 188,538 deaths as of September 5, 2020 [2]. The state of Washington, where the first American case of COVID-19 was detected, had 77,208 confirmed COVID-19 cases as of April 14, 2020 [3]. Given the rapid spread of COVID-19 and an associated mortality rate of 3.4% [4], countries like Italy and China have been forced to ration their limited health care resources, and there are concerns that the US may need to do so, as well [5]. Person-to-person transmission by asymptomatic and presymptomatic individuals during the up-to-14 day incubation period [6] may play a significant role in this pandemic [7-10].

Although data on the extent of hospital-acquired COVID-19 cases are unavailable, nosocomial infections have been shown to play a key role in propagating viral transmission in previous coronavirus outbreaks, such as the SARS outbreak in 2003 [11,12]. Because of the risk of exposure to SARS-CoV-2–infected patients and shortage of personal protective equipment (PPE) in the US as well as other countries [13-15], health care workers (HCW) have been disproportionately affected by the COVID-19 pandemic [16-18].

The aim of this study was to provide a quantitative analysis and model for predicting the impact of periodic COVID-19 testing for all emergency room staff as a possible alternate strategy to mitigate disease transmission in the health care setting, especially since PPE supplies are limited.

## Methods

### Data Sourcing

In order to model a hospital emergency department (ED) and a moderately affected patient population, we chose to base our model on Harborview Medical Center (HMC) and University of Washington Medical Center (UWMC) in King County, WA, because we had access to their ED staffing information. Because HMC and UWMC are two of many hospitals within the region, for the sake of simplicity, we assumed that the entire patient population from both these hospitals essentially lived in King County, WA.

In order to estimate the number of daily ED visits, we used the publicly available University of Washington Medicine Annual Financial Report for the Board of Regents meeting, which reported that 57,516 ED visits to HMC and 28,276 ED visits to UWMC were made during fiscal year 2018 [19]. Next, to estimate average daily ED visits, we divided this total number by 365 days, because medical emergencies happen daily regardless of holidays. Although it is possible that the rate of ED visits has changed because of COVID-19 symptoms, socio-behavioral changes, and public policies related to the COVID-19 pandemic, this information is currently not available to us.

The HMC ED currently employs 307 full-time HCW; these include 59 emergency medicine (EM) faculty physicians, 48 EM resident physicians, and 200 full-time equivalent registered nurses (RNs) and medical assistants (MAs). The UWMC ED currently employs 176 full-time HCW, including an estimated 44 EM faculty physicians, 28 EM resident physicians, and 104 full-time equivalent RNs and MAs.

### Initial Conditions

Our model is intended to be generalizable to any hospital in the United States; hence, we did not apply hospital-specific policies to our model and instead maintained the same constraints that many other US hospitals have.

**Figure 1:**
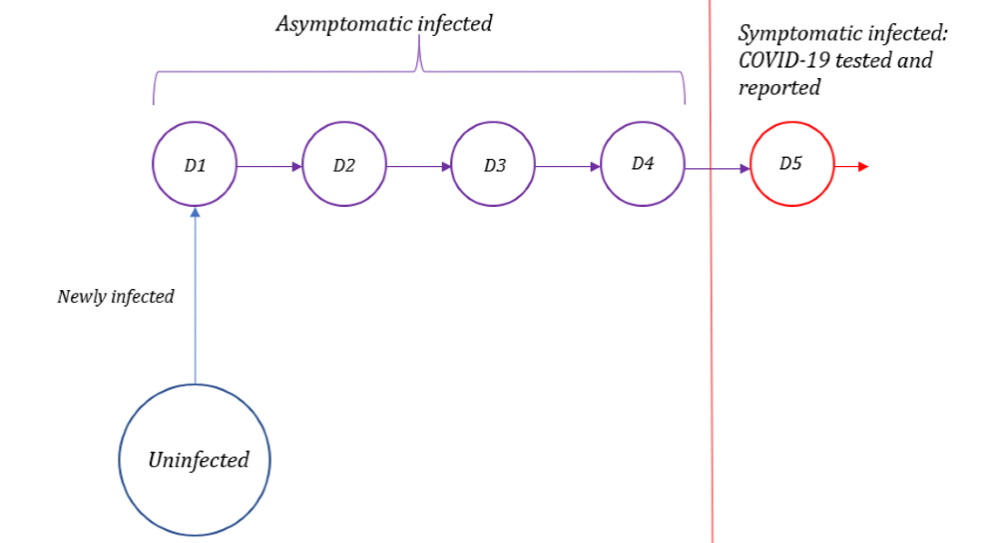
Timeline of infection for confirmed COVID-19 cases. After infection, an individual can transmit the infection to others but does not become symptomatic until day 5.

Owing to the incubation period of the virus, in addition to the current resource limitations in the US, COVID-19 testing is often not performed until symptoms become evident. Furthermore, laboratory test results for COVID-19 may not be available until patients have left the ED. To estimate the asymptomatic infected population, we examined the number of newly confirmed COVID-19 cases on each date, and we retroactively calculated the daily number of individuals that would have been in the presymptomatic incubation phase. The average incubation period for COVID-19 is approximately 5-6 days [20-22]. For our proposed model, we considered a shorter incubation period of 5 days, implying that symptoms begin on day 5 (Figure 1). Thus, for any time t, the number of asymptomatic infected individuals can be estimated by calculating the sum of new infections that were confirmed on t+1 to t+4, as follows:

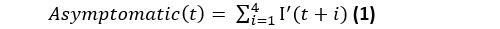


In other words, if an individual is symptomatic and tests positive for COVID-19 on any of the days between t+1 and t+4, then this individual can be assumed to be infected but asymptomatic on day t. Using data for King County, WA, collected up to April 5, 2020, we calculated that there were 685 asymptomatic individuals with COVID-19 in King County on April 1, 2020.

In order to determine the total number of infected individuals in King County on any given date, we added the number of publicly reported confirmed cases (as of April 1, 2020) to the number of asymptomatic infected cases that we calculated, from which we then subtracted the number of COVID-19–related deaths. To determine the total uninfected population, we subtracted the number of infected individuals and the number of COVID-19–related deaths from King County’s estimated 2019 population of 2,252,782 [23]. Subsequently, to determine the proportions of the living population that were infected and uninfected, we divided the total infected population and the total uninfected population, respectively, by the total living population.

Since the majority of patients and HCW reside locally, we assumed that their infection statuses would initially also be representative of that of the general population. Thus, to determine our initial values of the number of infected and uninfected patients and HCW/day, we multiplied our calculated proportions with the total number of ED patients/day and the total number of HCW in the ED. For HMC, the initial values thus calculated were 0.21 infected patients/day, 157.36 uninfected patients/day, 0.41 infected HCW, and 306.57 uninfected HCW. For UWMC, the initial values are 0.12 infected patients/day, 77.34 uninfected patients/day, 0.28 infected HCW, and 175.72 uninfected HCW.

Infected HCW were further subdivided into groups based on how long they had been infected. Because asymptomatic individuals with COVID-19 would continue to remain in the workforce, we also included infected HCW in the health care workforce for days 1-4 of their infection (during this period, they could likely infect other HCW and patients); these infected HCW were removed from the workforce on day 5, when they likely displayed symptoms. For our initial conditions, we uniformly divided the infected HCW into 4 groups: HCW on day 1 of infection (D1), day 2 of infection (D2), day 3 of infection (D3), and day 4 of infection (D4).

### Transmission Rate

To investigate the number of preventable infections of HCW from asymptomatic infected patients, we used a logistic model of transmission. The mathematical logistic model used described a dynamic population growth rate that is limited by a certain constraint such as population. In epidemiology, logistic models have been used successfully to model and predict past outbreaks [24,25].

In the equation below, *k* is the transmission constant, *M* is the total population size, 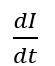 is the rate of change of infected population, and *I* represents the total infected population, including the asymptomatic infected population.

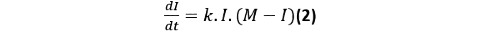



However, since our data is publicly sourced and case reports are available only on a daily basis, we use the discretized form of the logistical model, as follows:

I’(t) = k ∙ I(t) ∙ (M−I(t)) **(3)**

I’(t) is the rate of change of the infected population; hence, it can be observed that it is the difference between the infected population at time t+1 and time t.

I’(t) = I(t+1) − I(t) **(4)**

To calculate the transmission constant, we rearranged the previous equations to the following:

k = (I(t+1) − I(t)) / (I(t) ∙ [M−I(t)]) **(5)**

To determine the total infection spread, we need data for some known infected populations, both symptomatic and asymptomatic. For this purpose, we used data extracted from the Diamond Princess cruise ship [6], since the closed quarters of the ship approximate the clinical setting. Due to the isolated nature of the ship, health officials were able to test all individuals onboard the cruise ship for COVID-19, even if no symptoms were evident. Using the data at hand and the above equation, we can readily determine the transmission constant by dividing the number of new cases at time t+1 (with time measured in days) by the product of infected population at time t and the uninfected population at time t, which we calculated to be an average of k = 1.219e-4 new infections/person^2^.

We know the transmission rate for HCW would likely vary; however, we cannot ascertain whether it would be higher or lower, considering the fact that HCW would likely be in more intimate and close contact with patients than typical interactions between individuals on a cruise ship, and considering factors such as PPE usage by the HCW. Furthermore, the transmission rate varies by department and institution as well. Since we do not have an accurate transmission rate for the resource-limited clinical environment, we decided to model several different scenarios using 3 times the transmission constant (3.660e-4 new infections/person^2^) and one-third the transmission constant (4.067e-5 new infections/person^2^) calculated from the Diamond Princess cruise ship data.

To calculate the number of patient-to-HCW infections, HCW-to-patient infections, and HCW-to-HCW infections occurring in the ED, we adapted the logistic model to the following equation:

I_m_’(t) = k ∙ I_n_(t) ∙ U_m_(t) **(6)**

where I _m_’(t) refers to the new infections of a population *m*, k is the transmission constant, I__n_(t) refers to asymptomatically infected individuals of the group *n* transmitting the virus, and refers to uninfected individuals of the group *m* that is being newly infected. For instance, if I__m_’(t) represents new HCW-to-patient infections, then I__n_(t) would represent asymptomatically infected HCW, and U__m_(t) would represent uninfected patients presenting to the ED. These calculations would be repeated in our model for every day.

Assuming adequate inpatient beds are available, a number of patients leave the ED each day—this could either mean they were admitted to the hospital or that they were leaving the institution. On the other hand, a new batch of patients with characteristics representative of the general population would visit the ED each day. Therefore, the initial numbers of uninfected patients and infected patients that we used for our calculations remained constant. In reality, the number of infected patients presenting to the ED may be disproportionately higher than that in the general population, since completely healthy individuals without any acute illness or injury would not visit the ED.

**Figure 2:**
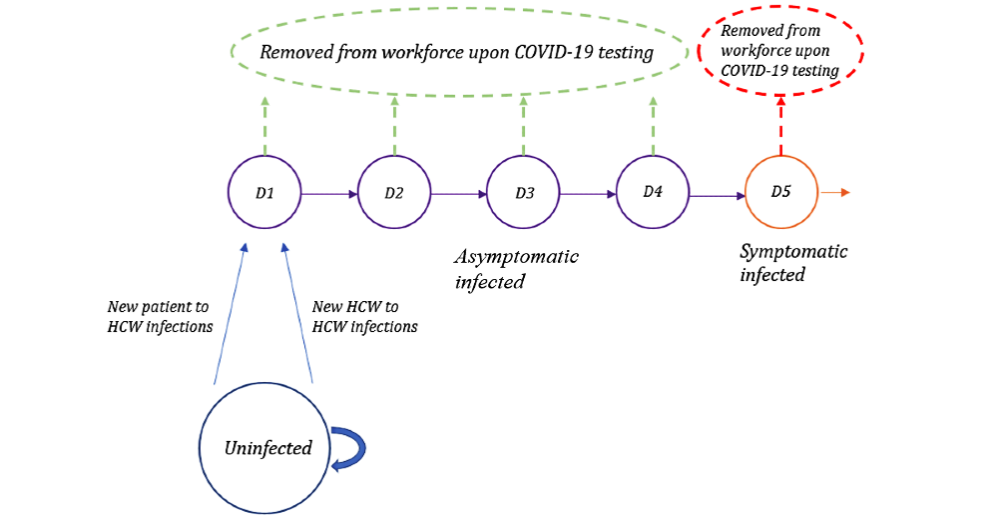
Markov chain for HCW. HCW who are uninfected on any given day can either remain uninfected or become newly infected (blue), at which point they would proceed to Day 1 of infection the next day. Individuals who are infected will proceed to the next day of infection (eg, D1, D2) with each passing day. Infected HCW are asymptomatic on days 1-4 (purple). On day 5 of infection, infected individuals begin showing symptoms (orange), at which point they are removed from this workforce. With COVID-19 testing conducted earlier, asymptomatic infected HCW who test positive may also be removed from the health care workforce earlier, on the day when COVID-19 test results are obtained (green). HCW: health care.

In addition, since the two hospitals were unlikely to have significant changes in their employment in the time period for which we were modeling, we designed a Markov chain to track their infection timelines (Figure 2). New HCW infections constituted the D1 group for the following day, and HCW in D1 would be moved to D2 the following day, HCW in D2 would be moved to D3 the day after, and so on.

### Periodic Testing

To simulate periodic COVID-19 testing of all HCW, we assumed COVID-19 test sensitivity of 100%, for the sake of simplicity. In reality, however, test sensitivity is likely to be lower and may vary based on how testing or sample collection is performed; our model can accordingly be adapted for other levels of sensitivity. Currently, there is insufficient data on COVID-19 testing to retrieve information on test sensitivity. On any given day that all HCW are tested, we would manually eliminate (sensitivity)*(number of infected HCW on each day) from each category. With an assumption of 100% sensitivity, this would mean that all infected HCW would be removed from the work force on the day the test was performed (Figure 2). In the case of weekly testing, we started this manual elimination process on day 6, and then repeated this process every 7 days. In the case of biweekly testing, we started the manual elimination process on day 13, and then repeated this process every 14 days.

## Results

Our model predicts that over the course of 180 days, 28,364 and 13,945 patients visited the ED in HMC and UWMC, respectively. Tables 1 and 2 show how the predicted numbers of COVID-19 infections in new patients and HCW vary with different transmission rates and frequencies of COVID-19 testing for HCW at both HMC and UWMC. At the baseline, with a transmission constant of 1.219e-4 new infections/person^2^, without routine COVID-19 testing of HCW, 1.914 HCW infections and 0.985 new patient infections would occur in HMC, and 0.505 HCW infections and 0.223 new patient infections would occur in UWMC. If COVID-19 testing of HCW was performed every 7 days (weekly), 1.802 HCW infections and 0.927 new patient infections would occur in HMC, which would be a 5.9% reduction in both HCW and new patient infections. Similarly, weekly COVID-19 testing for HCW in UWMC would result in 0.489 HCW infections and 0.215 new infections, which would yield a 3.1% reduction. If COVID-19 testing of HCW occurred every 14 days (biweekly), 1.873 HCW infections and 0.964 new patient infections would occur in HMC, which would yield a 2.1% reduction in both HCW and new patient infections. Biweekly COVID-19 testing in UWMC would result in 0.499 HCW infections and 0.220 new patient infections, which would yield a reduction of 1.1%.

**Table 1:** New patient infections with and without periodic COVID-19 testing for HCW at HMC and UWMC. Predicted numbers of new patient infections with various transmission rates and COVID-19 testing frequencies for HCW after 180 days within the emergency departments of HMC and UWMC. Percentages in parentheses represent the reduction in the number of infections at the given transmission rate with weekly (every 7 days) or biweekly (every 14 days) testing compared to the number of infections with no routine COVID-19 testing for HCW. HCW: health care workers; HMC: Harborview Medical Center; UWMC: University of Washington Medical Center.

COVID-19 transmission rate	COVID-19 testing frequency					
	HMC			UWMC		
	No testing	Weekly testing	Biweekly testing	No testing	Weekly testing	Biweekly testing
1.219e-4 new infections/person^2^	0.985	0.927 (5.92%)	0.964 (2.14%)	0.223	0.215 (3.17%)	0.220 (1.14%)
3.660e-4 new infections/person^2^	4.475	3.409 (23.81%)	3.906 (12.70%)	0.819	0.726 (11.32%)	0.773 (5.56%)
4.067e-5 new infections/person^2^	0.295	0.289 (1.77%)	0.292 (0.85%)	0.0699	0.0693 (1.0%)	0.0696 (0.48%)

**Table 2:** HCW infections with and without periodic COVID-19 testing for HCW at HMC and UWMC. Predicted numbers of new HCW infections with various transmission rates and COVID-19 testing frequencies for HCW after 180 days within the emergency departments of HMC and UWMC. Percentages in parentheses represent the reduction in the number of infections at the given transmission rate with weekly (every 7 days) or biweekly (every 14 days) testing compared to the number of infections with no routine testing for HCW. HCW: health care workers; HMC: Harborview Medical Center; UWMC: University of Washington Medical Center.

COVID-19 transmission rate	COVID-19 testing frequency					
	HMC			UWMC		
	No testing	Weekly testing	Biweekly testing	No testing	Weekly testing	Biweekly testing
1.219e-4 new infections/person^2^	1.914	1.802 (5.86%)	1.873 (2.11%)	0.505	0.489 (3.15%)	0.499 (1.13%)
3.660e-4 new infections/person^2^	8.596	6.582 (23.42%)	7.524 (12.47%)	1.850	1.643 (11.21%)	1.748 (5.50%)
4.067e-5 new infections/person^2^	0.573	0.563 (1.77%)	0.569 (0.84%)	0.159	0.157 (0.99%)	0.158 (0.47%)

With a transmission constant of 3.660e-4 new infections/person^2^, without routine COVID-19 testing of HCW, 8.596 HCW infections and 4.475 new patient infections would occur in HMC. The corresponding numbers for UWMC would be 1.850 HCW infections and 0.819 new patient infection. If COVID-19 testing of HCW was conducted weekly in HMC, 6.582 HCW infections and 3.409 new patient infections would occur, which is a 23% reduction in both HCW and new patient infections. In UWMC, weekly COVID-19 testing of HCW would result in 1.643 HCW infections and 0.726 new patient infection, which is a reduction of 11.2%. Biweekly COVID-19 testing of HCW (every 14 days) in HMC would result in 7.524 HCW infections and 3.906 new patient infections, which is a 12.47% reduction in HCW infections and a 12.7% reduction in new patient infections. Biweekly testing in UWMC would result in 1.748 HCW infections and 0.773 new patient infection, which is a 5.5% reduction.

For a lower transmission constant of 4.067e-5 new infections/person^2^, 0.573 HCW infections and 0.295 new patient infections would occur in HMC without routine COVID-19 testing of HCW. Similarly, 0.159 HCW infections and 0.0699 new patient infections would occur in UWMC. In the case of weekly COVID-19 testing of HCW in HMC, 0.563 HCW infections and 0.289 new patient infections would occur in HMC, which is a 1.7% reduction in both HCW and new patient infections. In UWMC, 0.157 HCW infections and 0.0693 new patient infections would occur, which is an approximately 1% reduction. In the case of biweekly COVID-19 testing of HCW in HMC, 0.569 HCW infections and 0.292 new patient infections would occur, which is an approximately 0.85% reduction in HCW infections and new patient infections. In UWMC, this would result in 0.158 HCW infection and 0.0696 new patient infection, which is an approximately 0.47% reduction in potential infections.

## Discussion

Our model shows that, within a hospital ED, periodic COVID-19 testing of HCW would reduce the rate of SARS-CoV-2 infection among ED personnel as well as new patients in the ED. As expected, the impact of periodic HCW testing varied with the transmission rate of SARS-CoV-2, with greater benefits observed when the transmission rates were higher.

Our model used the COVID-19 prevalence data for King County, WA, an area which is not as heavily affected by COVID-19 as many other places in the US, considering disease prevalence among patients visiting the ED. A higher COVID-19 prevalence in the patient population may result in higher patient-to-HCW disease transmission rates, in which case, periodic HCW testing would be more beneficial.

A limitation of our model is that we do not know the actual transmission rate in various hospital EDs; furthermore, transmission rates may vary widely between hospitals based on PPE supply, type of interactions with patients, and severity of illness (which also affects the types of procedures and therapies involved), and other factors. Additionally, the transmission rate may be different for different types of HCW; for instance, those who perform aerosolizing procedures such as intubation may be subject to a higher rate of transmission.

By changing the initial parameters, this model can be adapted for different ED visit rates, ED staffing numbers, levels of infection prevalence, transmission constants, and levels of testing sensitivity. Lower levels of testing sensitivity will lead to decreased utility in periodic HCW testing. In addition, our analysis was performed with the population characteristics of a county that is moderately affected by COVID-19. Currently, many regions of the US have a much higher COVID-19 prevalence, wherein periodic HCW testing would result in a greater potential benefit to prevent HCW infections.

Moreover, due to the current state of COVID-19 testing, US statistics on confirmed COVID-19 cases may not be the most reliable. Per CDC guidelines that were updated on March 24, 2020, at the time of writing this manuscript, laboratory testing for COVID-19 is only indicated for individuals who are not HCW nor first responders and have symptoms that are consistent with COVID-19 [26]. However, many individuals with COVID-19 may be asymptomatic or only have mild symptoms [27]. In addition, shortages of COVID-19 tests may affect the US statistics on COVID-19 cases, making them less reliable [28]. Therefore, the numbers for COVID-19 incidence and prevalence used in our model, which are based on official reports, may be erroneously low.

Of note, our model only includes ED staff in our numbers, but HCW from other specialties and departments also see patients in the ED. For instance, in many hospitals, non-EM physicians will see inpatient admissions in the ED, specialists may be consulted to see patients in the ED, and surgeons and anesthesiologists may be involved in cases of trauma resuscitations. Additionally, at some teaching hospitals, resident physicians in specialties outside of EM will also have EM rotations.

Given the uncertainty and unavailability of data regarding COVID-19, some of the numbers and factual assumptions in this model may be incorrect, which could affect the model’s predictions. To simplify calculations, this model assumes that COVID-19 infections are spread homogeneously throughout the state and that HCW freely interact with patients and all other HCW. Moreover, the model does not consider individual variation in COVID-19 incubation times. Ultimately, this model is intended to be a tool and an approximation, and it can be adapted to different health care settings or regions by varying the initial conditions.

## Abbreviations

ED: emergency department
EM: emergency medicine
HCW: health care workers
MA: medical assistant
RN: registered nurse
SARS: severe acute respiratory syndrome
PPE: personal protective equipment
